# Stimulus over expertise: what governs clinical visual attention in early health-vocational students

**DOI:** 10.3389/fmed.2026.1956106

**Published:** 2026-10-15

**Authors:** Burcu Yuksel, Begüm Çapa Tayyare, Nurullah Eryilmaz

**Affiliations:** 1 Kocaeli Vocational School of Health Services, Kocaeli University, Kocaeli, Türkiye; 2 Institute of Educational Sciences, Leuphana University, Luneburg, Germany

**Keywords:** 2×2 mixed design, areas of interest, clinical reasoning, eye tracking, health-professions education, perceptual expertise, visual attention

## Abstract

**Introduction:**

Eye tracking is increasingly used to investigate how health-profession students allocate visual attention to clinically critical regions of diagnostic and therapeutic images. However, whether disciplinary training produces a perceptual advantage for images within students' own fields remains unclear. This study examined whether students preferentially attended to critical regions in images from their own discipline compared with those from an unfamiliar discipline.

**Methods:**

A within-stimulus 2 × 2 mixed design was employed involving 34 Medical Laboratory Techniques (MLT) students and 29 Occupational Therapy (OT) students. Participants viewed clinical images from both disciplines while their eye movements were recorded. Within each image set, own-discipline and visiting viewers were compared on attention to predefined critical regions and related fixation and timing measures. The potential predictive roles of academic standing and cognitive and learning-style variables were also examined.

**Results:**

No consistent own-discipline advantage was observed across the eye-tracking measures. Attention to critical regions did not favour students from the corresponding discipline in either image set, and statistically significant differences, where observed, favoured visiting rather than own-discipline viewers. In contrast, image identity consistently exerted a substantial effect on visual attention across all conditions. Neither academic standing nor cognitive or learning-style variables predicted attention to critical regions.

**Discussion:**

The findings challenge the assumption that enrolment in a health-profession programme necessarily confers discipline-specific perceptual expertise. Instead, visual attention appears to be influenced more strongly by stimulus characteristics than by disciplinary background at this stage of training. These results suggest that discipline-specific perceptual expertise may develop through accumulated, deliberate clinical exposure rather than through programme enrolment alone.

## Introduction

Medical and health-professional work depends heavily on visual interpretation. Whether a laboratory technician inspecting a stained smear, an occupational therapist evaluating a patient's grasp during a functional task, or a radiologist reading a volumetric scan, practitioners must quickly and reliably locate clinically meaningful information within complex visual scenes. Eye tracking has become a leading method for making this otherwise invisible process explicit, because the location, duration, and sequence of fixations offer an objective window onto where attention is directed and, by inference, what information is prioritised (1, 2). A key advantage over self-report is that gaze metrics are physiological rather than introspective, and are therefore less vulnerable to the biases that affect subjective workload questionnaires (3, 4). The interpretive logic rests on the long-standing eye–mind assumption that fixation location and duration index the cognitive processing of the fixated information, while saccades, the rapid movements between fixations during which little new information is acquired, reposition the gaze (3, 5). In health-professional education specifically, eye tracking has been applied to characterise how learners attend to diagnostic and therapeutic images, to compare novices with experts, and to design feedback that makes effective scanning strategies teachable (3, 6). Reviews of this literature map particular metrics onto particular cognitive processes, with fixation count and duration, saccadic indices, and pupil diameter serving as the principal proxies for visual attention, perception, memory, and cognitive workload (3, 7–9). Integrated multimodal frameworks formalise this mapping further, relating fixation, saccade, and pupil parameters to information-seeking and cognitive load during task solving, and combining them with learner characteristics to model the learning process (10).

A recurring claim in this literature is that domain expertise reshapes visual attention. Experienced clinicians are repeatedly reported to distribute their gaze more efficiently, cover a broader set of clinically relevant areas of interest (AOIs), and reach critical regions faster than novices. In a recent scoping review of eye tracking in nursing simulation, expert–novice gaze differentiation was one of three dominant themes, with experienced nurses showing broader AOI coverage and more efficient scanning, while novices fixated longer on non-salient cues and were slower to locate key information (6). Comparable expert advantages have been described in radiology, where systematic viewing and rapid localisation of abnormalities distinguish more experienced from less experienced readers (11). Reviews of medical-image interpretation similarly report that experts and novices differ in the duration and number of fixations directed at diagnostically relevant AOIs, and that errors can be partitioned into scanning, recognition, and decision components according to whether and how long an abnormality was fixated (3). The implicit developmental model is that perceptual expertise accumulates with training and clinical exposure, gradually tuning attention towards the regions that matter.

This raises a question that is rarely tested directly: at what point in training does such disciplinary perceptual tuning emerge? Most expert–novice studies contrast individuals separated by years or decades of clinical experience. Far less is known about whether early-stage vocational students, who have completed foundational coursework but have limited hands-on clinical exposure, already show discipline-specific attentional advantages. The scoping review is explicit that expert-like gaze behaviour is associated with accumulated clinical experience rather than mere programme membership, and that, in the absence of a validated way to pre-classify learners by perceptual proficiency, clinical experience is typically used as a proxy for expertise (6). If perceptual expertise is genuinely a product of deliberate clinical practice, then students who differ in nominal discipline but not in accumulated expertise should not differ in how they attend to their ‘own’ versus an ‘unfamiliar’ image domain.

A second body of work cautions against over-interpreting eye-movement differences even when they are statistically reliable. Across multimedia-learning experiments, manipulations that strongly alter where and how learners look frequently fail to change what they learn. In a within-subjects study of instructor presence, a visible talking head sharply reduced fixation duration on the learning content, reduced the number of fixations on newly appearing content, and delayed time to first fixation, yet produced no difference in knowledge-test or picture-recognition performance, and the eye-movement effects did not mediate learning (12). Two studies of pointing and labelling in split-attention materials reached an analogous conclusion: mouse pointing and labelling changed fixation durations, fixation counts, and text–picture transitions, but these perceptual effects did not translate into learning gains, leading the authors to conclude that the manipulations influenced perceptual rather than cognitive processing (13). The general lesson is that attentional metrics and outcome measures are dissociable, and that attention is best treated as a process indicator rather than as a proxy for competence. This dissociation is intelligible within cognitive-load theory and the cognitive theory of multimedia learning, which hold that learning depends on how working-memory resources are allocated to intrinsic, extraneous, and germane processing rather than on the gross amount of looking a stimulus elicits (14–16). Consistent with this, a study of health-sciences undergraduates that monitored a self-regulated learning task with eye tracking found that prior knowledge did not predict indicators of cognitive load, and that data-driven clusters of learners emerged that were not explained by prior or acquired knowledge, underscoring that gaze-based load indices are shaped by factors beyond the learner variables most often assumed to drive them (7, 17).

A third strand of evidence concerns the interplay between cognitive load and visual attention, and the methodological fragility of eye-tracking indices. Work in medical image interpretation has shown that the time needed to locate a relevant structure is related to pupil dilation, a proxy for cognitive load. Importantly, overall test performance did not predict cognitive load in a relatively homogeneous sample of second-year medical students, a null result the authors attributed to insufficient discriminability within a single training cohort (11). Studies that combine physiological and ocular measures in authentic clinical settings document how readily eye-tracking metrics can be distorted: in trauma-resuscitation work, blink-rate estimates derived from gaps in gaze data reached implausible values of up to 200 blinks per minute, and pupillometry was compromised by ambient-light changes, leading the authors to discard these indices (18). In an anxiety study combining a dot-probe task with free-viewing eye tracking, the eye-tracking outcome failed to show a hypothesised effect that the response-time measure did show, a dissociation the authors traced in part to task design and to the well-documented unreliability of certain attentional indices (19). Together, these studies make two points central to the present work: cognitive load and stimulus characteristics shape attention powerfully, and raw eye-tracking counts must be scrutinised for processing artefacts before they are compared across groups. The pupillometry literature is especially instructive here. Task-evoked pupillary responses are reliable proxies for cognitive workload, but only after baseline correction, because absolute pupil size varies widely between individuals and is confounded by ambient lighting, viewing distance, and gaze direction (20–22). Reviews of clinical-simulation research accordingly recommend subtractive baseline correction, constant ambient lighting, and explicit reporting of calibration and area-of-interest definitions, and they caution that differences in sampling rate, accuracy, and processing algorithm limit comparability across studies (3, 20, 21).

The present study brings these strands together in a design that, to our knowledge, has not previously been applied to early vocational health students. Rather than contrasting experts with novices, we used a within-stimulus 2 × 2 mixed design: two student groups from different disciplines, Medical Laboratory Techniques (MLT) and Occupational Therapy (OT), each viewed the experimental image sets from both disciplines (Table 1). This yields, within each image set, a direct comparison between viewers for whom the images belong to their own discipline (the owning group) and viewers for whom the same images are unfamiliar (the visiting group). If early disciplinary training already tunes clinical visual attention, the owning group should attend more and more quickly to predefined critical regions than the visiting group, on the very same stimuli, so that holding stimulus features constant isolates the contribution of viewer discipline.

**Table 1:** The 2 × 2 mixed design (within-stimulus comparison).

Viewer group	Laboratory image set	Occupational-therapy image set
MLT students (*n* = 34)	Own discipline	Visiting
OT students (*n* = 29)	Visiting	Own discipline

MLT, medical laboratory techniques; OT, occupational therapy; N, number of participants.

We also asked whether academic variables (grade-point average, university-entrance score, year of study) and cognitive and learning-style variables (perceived workload, inhibitory control, Felder–Silverman dimensions) predict critical-region attention. Throughout, we treat eye-movement metrics as process indicators and avoid inferring competence from attention alone, consistent with the dissociations documented above (12, 13).

## Materials and methods

### Design

We used a 2 × 2 mixed design (viewer discipline between participants, image domain within participants), realised as a within-stimulus comparison (please see details of the participants in Table 2). The two factors were viewer discipline (MLT students; OT students) and image set (laboratory task set; occupational-therapy task set). Each participant viewed both image sets, yielding four design cells: MLT students viewing laboratory images (own discipline), MLT students viewing occupational-therapy images (visiting), OT students viewing occupational-therapy images (own discipline), and OT students viewing laboratory images (visiting). Within each image set, the critical contrast is between the owning and visiting groups on identical stimuli, which holds stimulus features constant and isolates any contribution of viewer discipline. We emphasise that the domain-ownership hypothesis was not pre-registered as an *a priori* expertise prediction; the mixed-design contrast is therefore reported as a planned but exploratory test, and inferential claims are accordingly conservative and FDR-controlled. To avoid any ambiguity about provenance: the data reported here come from a single study in which all four design cells were collected on the same apparatus and processed through one analysis pipeline. The two image sets are two conditions within that single study, not separately conducted or independently sourced datasets; where earlier drafts referred to ‘datasets’ this denoted the two image-set conditions of the one experiment, and we have standardised the wording accordingly.

**Table 2:** Participant characteristics.

Characteristic	MLT students	OT students
Number of participants	34	29
Age, years — mean (SD) [range]	19.5 (1.0) [17–21]	20.3 (1.6) [17–24]
Gender — women/men	29/5	26/3
Discipline	Medical Laboratory Techniques	Occupational Therapy
Year of study — first/second year	19/15	14/15
Vision	Normal or corrected-to-normal	Normal or corrected-to-normal
Colour-vision deficiency	None reported	None reported

### Participants

Participants were Medical Laboratory Techniques (MLT) students (*n* = 34) and Occupational Therapy (OT) students (*n* = 29) enrolled at a vocational school of health services. Both first- and second-year students were included, allowing year of study to serve as a proxy for accumulated training. Both year cohorts were deliberately represented in each discipline so that the two groups were matched on accumulated training as well as on nominal discipline: because the mixed-design contrast compares owning and visiting viewers on identical stimuli, including the same balance of first- and second-year students in both groups ensures that any own-discipline difference cannot be attributed to one group simply being further along in its programme. The two groups were demographically similar and typical of Turkish health-vocational cohorts, with a strong female majority. Among the MLT students, 19 were in their first year and 15 in their second, with a mean age of 19.5 years (SD = 1.0; range 17–21); 29 were women and 5 were men. Among the OT students, 14 were in their first year and 15 in their second, with a mean age of 20.3 years (SD = 1.6; range 17–24); 26 were women and 3 were men. Because both groups were overwhelmingly female (only 5 men in the MLT group and 3 in the OT group), gender was not examined as a predictor of the eye-tracking metrics: the marked imbalance would render any gender-based comparison statistically uninformative and prone to unstable estimates. Gender is therefore reported for descriptive completeness only, and the analyses below collapse across it. Because the vocational curriculum introduces clinical placement and discipline-specific vocational courses in the second year, year of study also indicated whether a participant had undertaken clinical placement and vocational coursework: all second-year students had done so, and no first-year students had, so these two variables coincided with year of study and were not analysed separately. All participants had normal or corrected-to-normal vision, and none reported a colour-vision deficiency relevant to the stimuli. Participation was voluntary, and informed consent was obtained. The study was conducted in accordance with the Declaration of Helsinki; ethics-committee approval details are provided in the Declarations.

### Stimuli and critical AOIs

Each image set comprised six clinical images (laboratory set: G1–G6; occupational-therapy set: six task images covering orthosis use, foot assessment, grasp, therapeutic devices/modalities, joint involvement, and supination). The images were authentic clinical photographs; some of them were taken from real patients and contain potentially identifiable information. For this reason, example stimuli are not reproduced here and the set is not deposited publicly (see Data Availability Statement). For each image, a critical area of interest (AOI) was defined *a priori* to mark the region a domain practitioner would need to inspect to interpret the image correctly. The critical region was identified by domain experts (clinicians in the relevant field). More than one person was involved in specifying and checking these regions, and disagreements were resolved by discussion to a consensus boundary; because the AOIs were established by expert consensus at the design stage rather than by independent parallel coding of a common set, we did not compute a formal inter-rater agreement statistic, which we note as a limitation of the AOI procedure. Critical-AOI coordinates were established at the task-design stage; “verification against the recorded gaze data” here refers to a spatial-registration check confirming that each AOI's coordinates fell on the intended region of the displayed stimulus and that the recorded gaze was correctly aligned to stimulus space — it does not mean that AOI boundaries were adjusted to where participants happened to look, which would have been circular; AOI boundaries were identical across viewer groups so that the owning and visiting groups were scored against the same critical region on each image. Defining AOIs *a priori* is the standard means of constraining the otherwise unmanageable volume of gaze data to permit meaningful comparison between individuals and conditions, and the appropriate size and shape of an AOI depend on the stimulus and the goals of the study; AOI size varied across images because the diagnostically relevant region differed in extent from image to image; in every case the boundary was drawn to enclose that region and was then held identical across the owning and visiting groups, so that any variation in AOI area is a property of the stimulus rather than of the group comparison; and, following recommended practice, AOI boundaries were drawn to cover the diagnostically relevant region and held constant across groups to support reproducible scoring (3, 7).

### Procedure

Participants were tested individually. After the eye tracker was calibrated (see below), each participant completed a short practice and instruction phase to familiarise them with the task and the display before the recorded trials began. The task was an active clinical one rather than free viewing: for every image, participants were instructed to identify what was clinically relevant or abnormal in the image, that is, to inspect it as they would when interpreting it in practice rather than merely looking at it. Each of the twelve images (six from each task set) was presented for a fixed viewing window of 20 s, during which eye movements were recorded; images were presented one at a time, and the analysis window for each image corresponded to this 20-second presentation. The fixed window was chosen so that every participant contributed the same amount of viewing time to each image, keeping exposure constant across owning and visiting viewers and across the two image sets. Because the task required participants to search for and evaluate clinically meaningful content, any domain-specific perceptual advantage would have had the opportunity to express itself within the recorded window.

### Apparatus and recording

Eye movements were recorded with an EyeLink Portable Duo at a sampling rate of 1,000 Hz. Stimuli were displayed at 1,920 × 1,080 pixels. To ensure consistency across the two image sets, all analyses used monocular (left-eye) fixations and applied a dwell-weighted treatment to fixation data. Ocular dominance was not assessed; the left eye was recorded as a fixed monocular convention applied uniformly to every participant in both datasets. Because the same eye was used throughout, this choice is orthogonal to the own-discipline versus visiting contrast and cannot bias the mixed-design comparison. Stimulus onset times were taken from the experimental presentation script and verified against the recorded message stream, so that per-image analysis windows were aligned with the actual appearance of each image.

### Eye-tracking measures

For each participant and image, we computed the following. Fixations and saccades were identified using the EyeLink system's built-in event parser, which classifies events from the eye-velocity and eye-acceleration signal using the manufacturer's default detection settings; we did not substitute a custom algorithm or alter the default thresholds. Before computing any measure, samples corresponding to blinks and to periods when gaze fell outside the display area were removed, so that only valid on-screen fixations contributed to the metrics. All reported measures were then derived from these cleaned, left-eye event sequences. Two of the metric families were expressed as ratios rather than raw counts to make them comparable across images and participants and robust to differences in total fixation number between cells: the distractor measure as a distractor/total-fixation ratio and the saccade measure as saccades per second (this is what is meant by the “normalised form” and the dwell-weighted treatment referred to above). Critical-AOI attention was the proportion of fixations within the critical AOI relative to total fixations on the image (a ratio metric). Time to first fixation (TTFF) was the latency from image onset to the first fixation within the critical AOI. Mean fixation duration within the critical AOI was taken as an index of processing at that location; longer fixations are conventionally associated with deeper or more effortful processing (23, 24), although longer dwell can equally reflect difficulty, uncertainty, ambiguity, or novelty rather than depth, and we interpret it with that ambiguity in mind. Two additional families, distractor fixations (fixations outside the critical AOI) and saccade counts, were computed only in normalised form (distractor/total-fixation ratio; saccades per second). The distractor family (fixations in regions outside the critical AOI, expressed as a proportion of total fixations) is closely related to critical-AOI attention and carries little independent information about attention to the diagnostically relevant region; it is therefore not treated as a separate outcome in any analysis, and the critical-AOI ratio is the primary attention measure throughout. The distractor and saccade families are retained only to document the processing inconsistency discussed in the Results: both are sensitive to how fixations are counted and spatially assigned, and both are excluded from inference for that reason.

### Reliability of measurement and scoring

Because the eye-tracking indices were extracted automatically by the EyeLink system and its analysis software, scoring did not involve human judgement, and inter-rater reliability is therefore not applicable: the critical AOIs were defined *a priori* as fixed coordinates and applied identically to every participant, so re-running the same pipeline on the same recordings reproduces identical values by construction. Measurement reliability rested on the recording protocol rather than on subjective coding. Before each session the tracker was calibrated and validated with the standard EyeLink procedure, and recording proceeded only once an acceptable validation was obtained; gaze was sampled at 1,000 Hz, and the same monocular channel, AOI definitions, and analysis windows were applied across all four design cells. Because owning and visiting viewers were scored against the same critical region on the same stimuli with the same algorithm, any residual measurement error is shared across the compared groups and cannot generate a spurious own-discipline difference. Consistency of the processing pipeline was further checked by verifying stimulus-onset alignment against the recorded message stream and by confirming that per-cell fixation totals matched the tabulated values, which is how the raw-count inflation reported in the Results was identified and excluded from inference.

### Academic and cognitive/style variables

Academic standing was indexed by grade-point average (GPA) and university-entrance score, with year of study (first vs. second) serving as a proxy for accumulated training. Grade-point average was the cumulative GPA recorded on the institutional 4.00-point scale, and the university-entrance score was each student's admission score from Türkiye's centralized national higher-education entrance examination, the single criterion on which placement into the programme is based; year of study was taken directly from enrolment records. Cognitive and learning-style variables comprised perceived workload (NASA-TLX global score), inhibitory control — assessed with the Stroop colour–word task, in which naming the ink colour of an incongruently printed colour word requires suppressing the more automatic reading response, so that performance indexes the capacity to inhibit a prepotent response — (Stroop correction count; the Stroop error count was constant at zero and therefore not analysed), and the four Felder–Silverman learning-style dimensions (active–reflective, sensing–intuitive, visual–verbal, sequential–global). Perceived workload was the NASA-TLX global score, computed as the mean of its six subscale ratings (mental, physical, and temporal demand, performance, effort, and frustration). Inhibitory control was indexed by the number of self-corrections in the interference (colour-word) condition of the Stroop task. Each Felder–Silverman dimension was scored from the 44 forced-choice items of the Index of Learning Styles, yielding a signed value from +11 to −11 that locates a respondent between the two poles of the dimension, with values near zero indicating a balanced preference. These variables were examined as potential predictors of critical-region attention and as indicators of group differences. The pairing of Felder–Silverman learning-style dimensions with eye-tracking and academic-performance measures follows recent work in a comparable Turkish health-student population, which combined the Felder–Soloman Index of Learning Styles with gaze metrics and grade-point average (25). Consistent with the data-dictionary cautions accompanying the dataset, the NASA-TLX values were on a raw rather than a 0–100 scale, and the Stroop range was narrow; results for these measures are interpreted conservatively.

### Statistical analysis

The principal analyses were trial-level linear mixed-effects models that retain the crossed structure of the design, in which each participant contributes observations to several stimuli and each stimulus is viewed by many participants. For each outcome we modelled the single-image observation (one row per participant per image) with crossed random intercepts for participant and for stimulus, so that non-independence arising from repeated observations within participants and within images is modelled explicitly rather than removed by averaging. The domain-ownership hypothesis was tested directly as the viewer-discipline  ×  image-domain interaction (equivalently, the own-versus-visiting contrast), which asks whether the discipline difference reverses between the two image sets as an expertise account would require. Models were fitted by restricted maximum likelihood; we report fixed-effect estimates with their standard errors and the partition of variance between participants, stimuli, and residual. Mixed-effects models were fitted using standard mixed-model software for the R statistical environment; analysis scripts are available from the corresponding author (see Data Availability Statement). Because the images seen in the two sets are distinct stimuli, the stimulus random effect is specified over all twelve images. As complementary, more conservative checks that make no distributional assumptions, we also ran distribution-free tests on participant-level summaries, appropriate because the per-participant metrics were bounded ratios and latencies with small, unequal cell sizes for which normality could not be assumed. Domain-ownership contrasts (owning versus visiting viewers) were tested within each image set with two-sided Mann–Whitney U tests. Because Mann–Whitney U is a rank-based test, we quantified the magnitude of each contrast with the matched rank-based effect size, the rank-biserial correlation r, computed directly from the U statistic as r = 1−2U/(n₁n₂); by this convention r reported here is signed so that a positive value indicates the owning group had the lower critical-region engagement (or slower latency), that is, an effect in the direction opposite to the expertise hypothesis. Rank-biserial r is interpreted with the conventional thresholds of approximately 0.1, 0.3, and 0.5 for small, medium, and large effects. We had additionally reported Cohen's d as a familiar standardized-mean-difference descriptor, but because it presupposes the interval-scale, equal-variance assumptions that motivated the non-parametric tests in the first place, the rank-biserial correlation is the effect size on which interpretation should rest, and d is retained only as an auxiliary indication of the raw mean separation. Image-level effects were tested with Friedman tests across the six images, with Kendall's W as the accompanying rank-based effect size; associations with academic and cognitive/style variables used Spearman rank correlations. Missing data were handled by analysing all available observations without imputation: an image contributed to a given outcome only when that outcome could be computed for it (for the critical-AOI ratio this required a non-zero total-fixation count on the image), so the number of trials entering each model differed slightly across outcomes, and a small number of participants for whom only one image set was usable contributed to the analysis of that set alone. Because the mixed models use all valid trials rather than complete cases at the participant level, no participant was excluded listwise. One aspect of missing data requires specific mention. Time to first fixation is defined only when the critical AOI is fixated within the viewing window; on trials where a participant never fixated the critical region (about 19% of trials overall), time to first fixation is undefined and was recorded as such. These censored observations were not imputed and did not enter the time-to-first-fixation analysis, so that outcome is computed only over trials on which the critical region was fixated. This is a listwise treatment of censored latencies, and, as we note in the Limitations, it can bias the time-to-first-fixation results because the excluded non-fixation trials are not missing at random. For the multiplicity correction, a “family” was defined as the set of related tests addressing one question: the domain-ownership contrasts across the trustworthy outcomes and image sets formed one family, the image-level tests another, and the associations with academic and cognitive/style variables another; false-discovery-rate control was applied within each family. Across each family of related tests, *p*-values were adjusted with false-discovery-rate (Benjamini–Hochberg) control, and a two-sided *α* of 0.05 was used throughout.

## Results

### No domain-ownership advantage in critical-region attention

The central test of the study is whether the owning group attended more to the critical AOI than the visiting group on identical stimuli. It did not (Table 3). In the laboratory image set, MLT students (own discipline) directed 15.2% of their fixations to the critical AOI, compared with 17.3% for the visiting OT students; this difference was not significant (*p* = 0.25, r = −0.17; d = −0.40) and, if anything, favoured the visiting group. In the occupational-therapy image set, OT students (own discipline) directed 14.6% of fixations to the critical AOI versus 17.0% for the visiting MLT students; this difference reached nominal significance (*p* = 0.03, r = −0.33; d = −0.53) but in the direction opposite to the expertise hypothesis, again favouring the visiting group. Across all twelve image-by-set ownership comparisons, none survived FDR correction. Figure 1 shows the image-by-image owning-versus-visiting comparison; the owning group does not consistently exceed the visiting group on any image.

**Table 3:** Domain-ownership comparisons on trustworthy ratio and time metrics, by image set.

Metric	Image set	Own-discipline mean ± SD (n)	Visiting mean ± SD (n)	p	Cohen's d	r (rank-biserial)
Critical-AOI attention (ratio)	Laboratory (MLT)	0.152 ± 0.049 (34)	0.173 ± 0.056 (29)	0.252	−0.40	−0.17
Critical-AOI attention (ratio)	Occupational therapy (OT)	0.146 ± 0.042 (29)	0.170 ± 0.047 (34)	0.026	−0.53	−0.33
Mean fixation duration (ms)	Laboratory (MLT)	188.3 ± 48.6 (34)	187.7 ± 49.7 (29)	0.836	0.01	+0.03
Mean fixation duration (ms)	Occupational therapy (OT)	256.3 ± 50.0 (29)	246.4 ± 50.6 (34)	0.366	0.20	+0.13
Time to first fixation (ms)	Laboratory (MLT)	4,621.7 ± 1,871.4 (34)	3,217.5 ± 1,626.9 (29)	0.002	0.80	+0.46
Time to first fixation (ms)	Occupational therapy (OT)	3,157.5 ± 1,688.8 (29)	3,149.8 ± 1,679.9 (34)	0.972	0.00	0.00

Effect sizes are reported as the rank-biserial correlation (r), the effect size matched to the Mann–Whitney U test; Cohen's d is retained as an auxiliary descriptor. Own-discipline (owning) versus visiting viewers, participant-level means averaged across the six images; Mann–Whitney U tests with the rank-biserial correlation r as the matched effect size (Cohen's d shown for reference). Negative d denotes higher values for the visiting group. No comparison survived false-discovery-rate correction across the twelve image-by-set tests; the one nominally significant critical-AOI difference (occupational-therapy set) and the one significant time-to-first-fixation difference (laboratory set) both run opposite to the expertise hypothesis.

**Figure 1:**
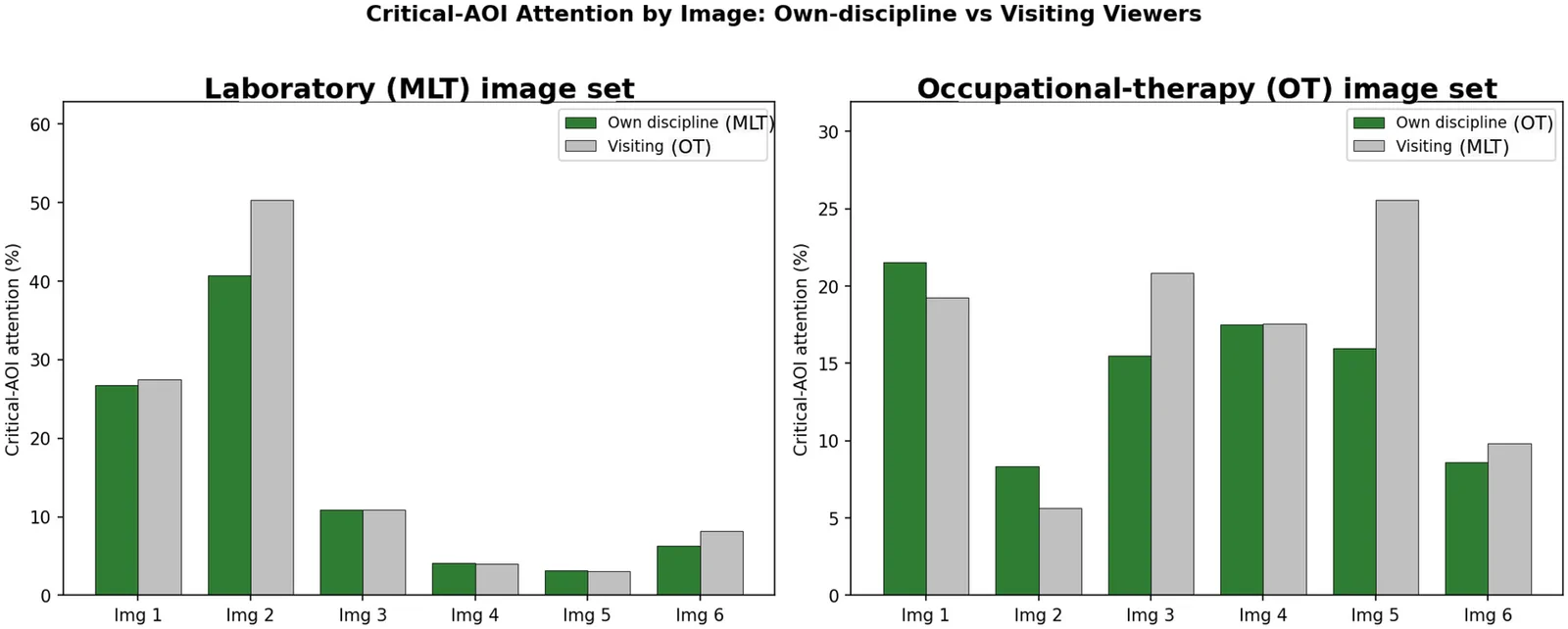
Critical-AOI attention by image for owning versus visiting viewers, in each image set. Bars show the proportion of fixations on the predefined critical AOI. No image shows a reliable advantage for the owning (own-discipline) group; where differences occur they tend to favour the visiting group.

The trial-level mixed-effects models, which are the primary test of the ownership hypothesis, confirmed this pattern while making the underlying structure explicit. Modelling the single-image critical-AOI ratio with crossed random intercepts for participant and stimulus, the viewer-discipline  ×  image-domain interaction — the direct test of a domain-ownership advantage — was not significant (b = −0.043, *p* = 0.074), and the equivalent own-versus-visiting contrast was, if anything, slightly negative (b = −0.021, SE = 0.012, *p* = 0.077), meaning owning viewers attended no more, and marginally less, to the critical region than visiting viewers. The same models were decisive about where the variance lay: stimulus identity accounted for about 68% of the systematic variance in critical-AOI attention, whereas the participant random effect accounted for essentially none (near 0%), with the remainder residual. The two time-and-duration outcomes told the same story. For mean fixation duration, neither the interaction (b = 10.0 ms, *p* = 0.52) nor the own-versus-visiting contrast (b = 0.7 ms, *p* = 0.93) approached significance, and again the variance was dominated by stimulus (about 66%) rather than participant (about 2%). For time to first fixation, the only outcome with a significant ownership term, the effect ran opposite to the expertise prediction: owning viewers were slower, not faster, to first fixate the critical region (interaction b = 1,490 ms, *p* = 0.015; own-versus-visiting b = 790 ms, *p* = 0.010), with stimulus again explaining about 66% of the variance and participants about 2%. Across all three trustworthy outcomes, then, the model that directly represents the crossed participant-by-stimulus design reproduces the null ownership result, locates almost all systematic variation at the level of the stimulus rather than the viewer, and — where an ownership effect reaches significance — finds it in the direction opposite to disciplinary expertise.

The same null is evident when the four cells are arranged as a 2 × 2 ownership matrix (Figure 2). If disciplinary training tuned attention, the diagonal cells (each group viewing its own discipline's images) should show closer critical-AOI attention than the off-diagonal cells. They do not; the diagonal is not elevated relative to the off-diagonal, confirming the absence of an own-discipline advantage at the group mean level.

**Figure 2:**
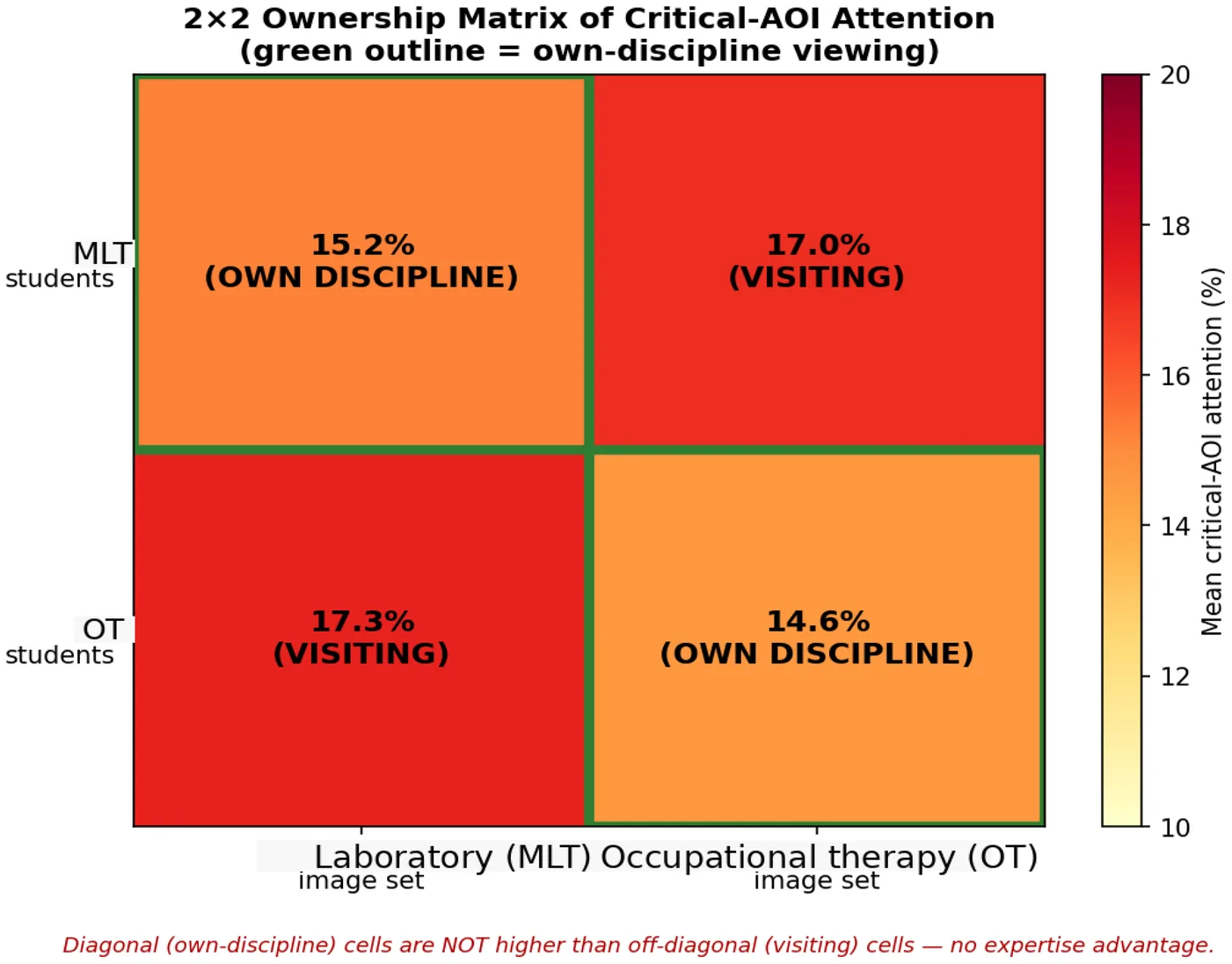
Two-by-two ownership matrix of critical-AOI attention. Rows are viewer discipline, columns are image set; diagonal cells (green outline) are own-discipline viewings. Own-discipline cells are not higher than visiting cells, contrary to the expertise hypothesis.

### Depth of processing and first-fixation latency show no ownership advantage

Mean fixation duration within the critical AOI did not differ between owning and visiting groups in either image set (laboratory set: 188.3 vs. 187.7 ms, *p* = 0.84; occupational-therapy set: 256 vs. 246 ms, *p* = 0.37), indicating that, when participants looked at the critical region, owning and visiting viewers processed it to a comparable depth. Time to first fixation likewise provided no support for an ownership advantage. In the laboratory set, owning viewers were in fact slower to first fixate the critical AOI than visiting viewers (4,622 vs. 3,218 ms, *p* = 0.002, r = 0.46; d = 0.80), the opposite of an expertise effect; in the occupational-therapy set, the two groups were indistinguishable (3,157 vs. 3,150 ms, *p* = 0.97). Thus, the only reliable difference on a trustworthy time metric ran counter to the expertise hypothesis.

### Image identity, not viewer discipline, governs attention

In sharp contrast to the absence of ownership effects, image identity exerted a large and consistent influence on attention across all cells. Friedman tests across the six images were significant in all four cells (all *p* < 0.01), with the strongest image effects in the laboratory-image cells (Kendall's W = 0.68 and 0.66) and smaller but still highly reliable effects in the occupational-therapy-image cells (W = 0.24 and 0.21); full statistics are given in Table 4. In other words, which image a participant viewed was a far stronger determinant of attention allocation than whether the image belonged to the participant's own discipline. This pattern is precisely what would be expected if attention at this training stage is driven by bottom-up stimulus features and task-general salience rather than by discipline-specific perceptual schemata.

**Table 4:** Image-identity effects on critical-AOI attention within each design cell (friedman tests across the six images, with kendall's W as effect size).

Design cell	n	Friedman chi-square	p	Kendall's W
MLT students/Lab images (own)	33	112.1	<1e−21	0.679
OT students/Lab images (visiting)	29	95.7	<1e−18	0.660
OT students/OT images (own)	27	32.0	<1e−5	0.237
MLT students/OT images (visiting)	19	19.9	0.001	0.209

Image identity is a large and consistent determinant of attention in every cell, in contrast to the absent domain-ownership effects in Table 3.

### Academic and cognitive/style variables do not predict critical-region attention

Neither academic standing nor cognitive or learning-style variables predicted attention to clinically critical regions. Grade-point average was essentially uncorrelated with critical-AOI attention (Spearman rho ≈ 0.00–0.04; no comparisons survived FDR correction), as was university-entrance score (rho ≈ −0.11 to −0.25). Year of study did not differentiate attention in either discipline (MLT: *p* = 0.55; OT: *p* = 0.88), indicating that an additional year of vocational training did not measurably sharpen attention to critical regions. Among the cognitive and learning-style measures, perceived workload (NASA-TLX), inhibitory control (Stroop correction count), and all four Felder–Silverman dimensions showed no group differences (all *p* > 0.16). Figure 3 summarises the analyses of academic variables; none of the relationships approached significance.

**Figure 3:**
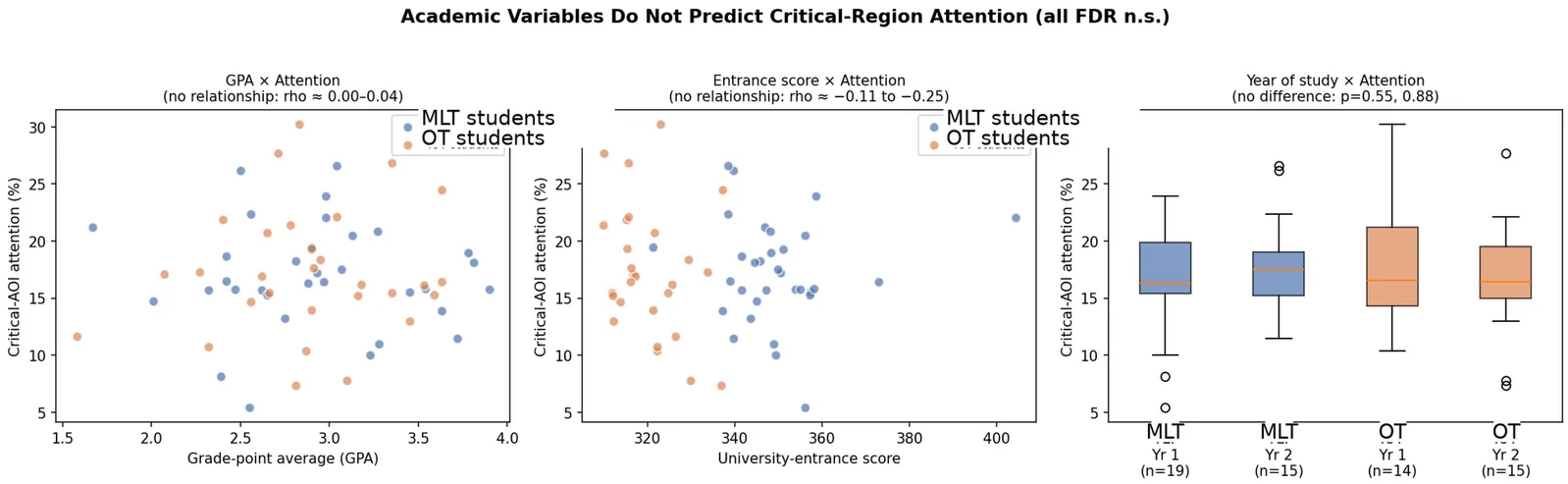
Academic variables and critical-region attention. Grade-point average, university-entrance score, and year of study are each plotted against critical-AOI attention. No variable predicts attention, indicating that academic standing and one additional year of training do not sharpen attention to clinically critical regions.

### Raw-count ‘effects’ are measurement artefacts

Two apparent group differences emerged on raw-count metrics, and both were traced to the processing artefact described in the Methods rather than to behaviour. In the occupational-therapy set, saccades-per-second appeared to differ markedly between the owning and visiting groups (d = 4.94), and the distractor ratio likewise (d = −10.38). Effect sizes of this magnitude are not psychologically plausible; a Cohen's d of around 5, let alone 10, does not occur for a genuine attentional contrast and is a hallmark of a scaling or processing difference. Inspection confirmed the cause: the cell in which OT students viewed their own images carried roughly twice the total fixation count of the other cells (≈ 3,026 vs. 1,347–1,505), and both ‘effects’ appeared only in that image set, not in the laboratory set. A real group difference would be expected to appear in both sets; its restriction to the inflated cell identifies it as an artefact of that cell's processing pipeline. Critically, the metric on which our main conclusions rest, the critical-AOI ratio, is empirically stable across the four cells, including the affected one (cell means 0.146–0.173), and its conclusions are independently reproduced by the trial-level mixed-effects models. We do not claim that expressing a metric as a ratio makes it immune to processing differences in general — indeed the distractor ratio, which shares the same ratio form, is badly distorted in one cell — because altered fixation parsing can change the number, duration, and spatial assignment of fixations non-proportionally. What we rely on is the observed stability of the critical-AOI measure and its corroboration by the mixed-effects analysis, not a general immunity of ratios. Figure 4 displays the trustworthy ratio and time metrics on a common effect-size axis, with the exact *p*-value beside each marker; these metrics cluster around zero, while the inflated raw-count metrics and the descriptive-only pupil metric are excluded from the figure for the reasons given above.

**Figure 4:**
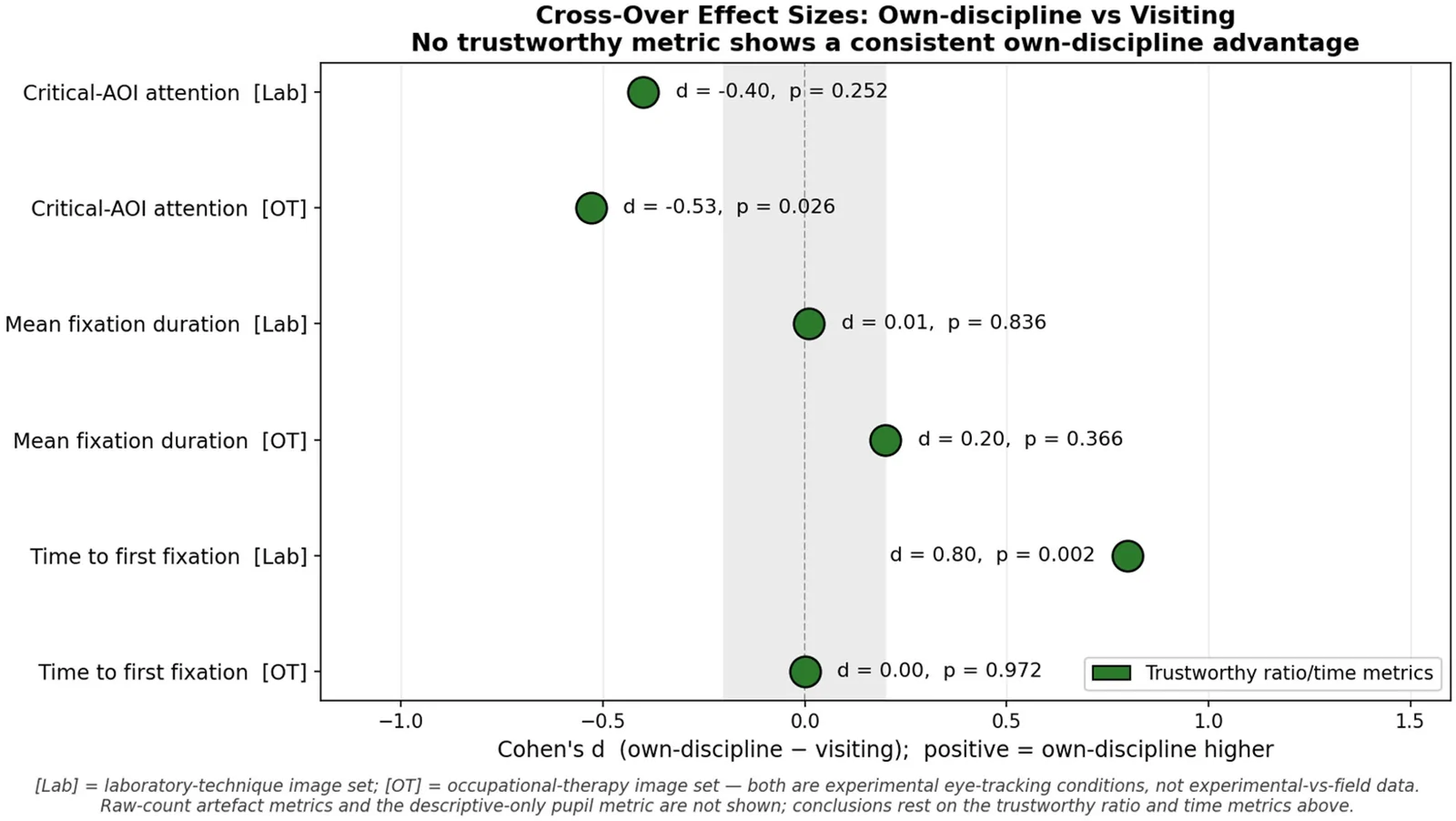
Mixed-design effect sizes (Cohen's d, own-discipline minus visiting) for the trustworthy ratio and time metrics, with the exact *p*-value shown beside each marker. [Lab] and [OT] denote the laboratory-technique and occupational-therapy image sets respectively; both are experimental eye-tracking conditions. The two raw-count metrics (saccades per second and distractor ratio) and the descriptive-only pupil metric are excluded from inference and omitted here, so that the axis shows only the trustworthy ratio and time metrics. No trustworthy metric shows a consistent own-discipline advantage, and the one nominally significant time metric (time to first fixation, laboratory set) runs opposite to the expertise hypothesis*.*

Taken together, the trustworthy metrics converge on a single conclusion: there is no own-discipline advantage in clinical visual attention at this training stage, and image identity dominates.

### Summary of findings

Five independent axes yielded null or anti-expertise results: domain ownership, year of study, grade-point average, university entrance score, and cognitive/style variables. A single factor, image identity, produced large and consistent effects. That several independent nulls coincide with equivalent data quality across cells and a strong stimulus effect points to a well-controlled result rather than an underpowered one.

## Discussion

Using a within-stimulus 2 × 2 mixed design, we asked whether early vocational health students already show a discipline-specific advantage in attending to clinically critical image regions. They did not. Across all trustworthy metrics, students attended to the critical regions of their own discipline's images no more, and sometimes less, than students for whom those images were unfamiliar. Meanwhile, image identity exerted a large and uniformly reliable effect, and neither academic standing, year of study, nor cognitive style predicted attention to critical regions. The most parsimonious account is that, at this stage of training, clinical visual attention is governed by stimulus properties and task-general salience rather than by disciplinary perceptual expertise.

### Perceptual expertise as an outcome of accumulated practice, not enrolment

This pattern aligns with the developmental model suggested by the expert–novice literature. The nursing scoping review concluded that expert-like gaze behaviour, broader AOI coverage, more efficient scanning, and faster localisation of critical cues are associated with accumulated clinical experience, and explicitly noted that, lacking a validated way to pre-classify learners by perceptual proficiency, researchers typically use clinical experience as a proxy for expertise (6). Our participants differed in nominal discipline but not in accumulated clinical expertise: both groups were early vocational students with limited hands-on exposure. On that reading, the absence of an own-discipline advantage is not a surprising failure but the expected result. The finding that one additional year of study did not sharpen critical-region attention reinforces this interpretation: programme membership and modest accrued coursework are not, by themselves, sufficient to install the attentional signatures of expertise. The wider medical-education literature describes the same trajectory from a different angle: expert–novice differences in fixation duration, dwell time, and the efficiency of search within diagnostically relevant AOIs are repeatedly observed when experience genuinely differs, with novices prone to scanning errors and experts to faster, more targeted localisation (3). The relevant contrast in those studies is accumulated practice, not nominal field; our within-stimulus design removes the stimulus confound that usually accompanies discipline comparisons and shows that, absent that experience gap, nominal discipline alone does not reproduce the expert signature. Direct evidence that the relevant advantage is experience-bound and domain-specific comes from pupillometric work with emergency-medicine trainees: physicians showed reduced task-evoked cognitive load relative to novices only when answering questions within their own field, with no group difference outside it, indicating that the cognitive efficiency conferred by expertise does not transfer to unfamiliar domains (26). That two groups separated by years of training differed only on home-domain material, whereas our two groups separated only by nominal discipline did not differ at all, is exactly the pattern expected if disciplinary tuning tracks accumulated practice rather than enrolment.

The radiology and medical imaging literature offers a converging precedent. In a study of second-year medical students interpreting volumetric images, overall test performance did not predict cognitive load, a null result the authors attributed specifically to the homogeneity of a single training cohort, which lacked the spread of expertise needed to reveal performance-linked differences (11). Our sample is homogeneous in precisely the relevant sense: all participants are early in training. The recurrence of this homogeneity-driven null across studies and metrics strengthens the case that disciplinary perceptual tuning is a later developmental achievement, and that cross-sectional contrasts among early students are unlikely to reveal it. A study of health-sciences undergraduates points the same way from the learner side: neither prior knowledge nor post-task knowledge scores predicted gaze-based indicators of cognitive load, and the behavioural clusters that emerged were not explained by those knowledge variables, implying that the learner attributes most often assumed to organise attention are weak determinants of it at this stage (7). Our null on grade-point average, university-entrance score, and year of study is the analogous result in a 2 × 2 mixed design.

### Stimulus dominance and the dissociation of attention from competence

The strong image-identity effect matters because it shows that our null is not a floor effect or a failure of measurement sensitivity: the same metrics that detected no ownership effect also detected very large stimulus effects. Attention was being driven robustly by something, just not by viewer discipline. This is consistent with a substantial body of multimedia-learning research showing that bottom-up stimulus features and design choices powerfully steer fixations, dwell time, and time to first fixation (27, 28). A well-established mechanism for this steering is bottom-up visual salience: low-level image properties such as local luminance and intensity, chromatic contrast and colour, edge and orientation contrast, and spatial frequency automatically attract gaze independently of a viewer's goals or expertise, and formal salience-map models predict fixation locations from these features alone (29). In our stimuli, such properties differed markedly from image to image but were, by design, identical for owning and visiting viewers of the same image, which offers a concrete account of why image identity dominated attention while viewer discipline did not: the same salient regions drew the gaze of both groups. Differences between images in contrast, colour saturation, clutter, and the visual clarity of the critical region are therefore the most plausible proximal drivers of the large image-identity effects we observed. Within cognitive-load and multimedia-learning theory, this is expected: where attention lands is jointly determined by the intrinsic complexity of the material and by extraneous, design-driven features of its presentation, so that strong stimulus-level variation in fixation patterns can arise without any corresponding difference in the learner's knowledge or in germane processing (14, 15, 30). It also fits the repeated finding that strong effects on attention need not imply effects on competence. As detailed in the Introduction, manipulations that strongly alter fixation duration, fixation counts, and time to first fixation can nonetheless leave learning outcomes unchanged, affecting perceptual rather than cognitive processing (12, 13). These dissociations warn against reading attentional allocation as a direct index of clinical competence, even though cognitive overload itself is well documented to impair performance in high-stakes clinical work (31). In our data, the inference runs the other way but rests on the same principle: the absence of an attentional difference between owning and visiting viewers should not be over-interpreted as evidence about underlying knowledge, only about where attention was deployed on these stimuli at this stage.

This caution has a direct methodological corollary that shaped our analysis from the outset. Because attentional metrics are process indicators rather than competence measures, and because they can be derived in circular ways, we were careful not to validate an attention-based proxy against other attention measures. Where the original datasets contained derived ‘attention proxy’ indices, we did not correlate them with the fixation metrics that generated them, since such a correlation would be self-fulfilling.

This mirrors a recurring theme in the eye-tracking methods literature: raw ocular indices are fragile and must be scrutinised before they are trusted. In trauma-resuscitation work, blink-rate estimates derived from gaps in gaze data reached up to 200 blinks per minute and were discarded as nonsensical. Pupillometry was abandoned because ambient-light changes overwhelmed the cognitive-load signal (18). In an anxiety study, the eye-tracking outcome failed to reveal an effect detected by the response-time measure, a dissociation the authors partly attributed to the known unreliability of certain attentional indices and to task design (19). The clinical-simulation methods literature makes the same point about raw ocular indices more generally: several require substantial correction and controlled recording conditions before they can be interpreted, which is why we based our conclusions on ratio and time metrics that are robust to such artefacts rather than on raw-magnitude indices (20–22). The nursing scoping review concluded its methodological appraisal by calling for standardised protocols for calibration, AOI definition, and reporting, precisely because differences in sampling rate, accuracy, and processing algorithms limit comparability across studies (6); reviews of eye tracking in medical education likewise stress theory-driven metric selection, careful calibration, and methodological triangulation with non-ocular measures as preconditions for trustworthy inference (3, 32).

### Educational implications

If disciplinary perceptual expertise is not yet present in early vocational students, curricula should not assume it. The data suggest that simply enrolling students in a discipline and advancing them by a year does not, on its own, tune their attention to clinically critical regions. This argues for explicit, deliberate training in visual-scanning strategies rather than reliance on incidental acquisition. The nursing simulation literature points to a workable mechanism: defining scenario-specific critical AOIs, establishing an expert benchmark scan, and using gaze replays and heatmaps in debriefing to make tacit scanning rules explicit and teachable (6). Because our students’ attention was dominated by stimulus salience rather than clinical relevance, interventions that direct attention to diagnostically critical regions and contrast a learner's gaze trace with an expert benchmark may be especially valuable early in training. At the same time, the dissociation literature (12, 13) warns that changing where students look will not automatically change what they learn; attention-directing interventions should therefore be paired with activities that promote deeper processing, and their effects should be evaluated against competence outcomes, not gaze metrics alone. Because gaze location alone cannot disclose whether a fixation reflects efficient recognition or unresolved difficulty, such interventions are best evaluated using methodological triangulation, combining eye tracking with think-aloud, performance, or other measures, as recommended for medical-education eye-tracking research generally (3).

### Limitations

Several limitations qualify these conclusions. First, and most importantly, the mixed-design domain-ownership contrast was planned but exploratory; no *a priori* expertise hypothesis was pre-registered, and the analysis is therefore best read as a well-controlled test of an assumption rather than as confirmation of a prediction. Second, the design measured visual attention but not behavioural accuracy: we did not collect participants’ clinical judgements about the images, so we cannot test whether attention to critical regions is necessary or sufficient for correct interpretation. This is a meaningful gap, because the dissociation literature shows that attention and performance can come apart, and a complete test of the perceptual-expertise question would require linking gaze to decisions. Third, although the task was an active clinical one (participants were asked to identify what was clinically relevant or abnormal in each image), viewing was bounded by a fixed 20-second window. This has two consequences. A participant scored as never fixating a critical region within the window might have done so given more time, so ‘never fixated’ should be read as ‘did not fixate within the 20-second analysis window’. This non-fixation was not negligible and, importantly, was not evenly distributed across conditions: the critical region was never fixated on about 19% of trials overall, ranging from roughly 9% in the cell where occupational-therapy students viewed their own images to about 26% in the cell where they viewed the laboratory images. Because these censored trials were dropped rather than modelled, the time-to-first-fixation means are computed over different, self-selected subsets in different cells — only the participants who did fixate the region — and the unequal censoring rates could bias the between-group time-to-first-fixation comparison in either direction. The time-to-first-fixation results should therefore be read with particular caution; a censoring-aware analysis (for example, modelling the probability of fixating alongside the latency, or treating unfixated trials as right-censored) would be needed to estimate these latencies without that bias, and we did not undertake one here. More substantively, a bounded, single-image inspection task may not fully engage the extended, self-paced diagnostic reasoning through which disciplinary perceptual expertise is thought to operate; a domain advantage that emerges only over longer, decision-driven examination could therefore go undetected here, and our null is most safely read as applying to early-timescale attentional deployment during constrained clinical inspection rather than to clinical interpretation in full. Fourth, the cognitive and learning-style measures carried documented scale limitations (raw-scale NASA-TLX; constant-zero Stroop error; narrow Stroop range), and the Felder–Silverman instrument is itself the subject of psychometric debate, so the null results on these measures should be read as consistent with, rather than definitive of, an absence of association. More generally, the academic and cognitive-style variables differ in their measurement properties: grade-point average and the entrance score are continuous, finely graded criteria, whereas the workload, Stroop, and learning-style measures are coarser and, as noted, scale-limited in this sample. The uniformly null associations should therefore be interpreted in light of these differing measurement criteria — the finding is that none of these indicators, at the resolution available here, tracked attention to critical regions — rather than as equally powered tests across variables. A further limitation concerns the raw-count metric families. Although the study was a single 2 × 2 experiment collected and analysed with one pipeline, a processing inconsistency inflated the raw fixation count in one design cell (the cell in which occupational-therapy students viewed their own images), producing the implausibly large ‘effects’ on the distractor and saccade-count families that we identified and excluded from inference. We emphasise that this affects only the raw-count metrics. The measures on which every substantive conclusion rests — the critical-AOI attention ratio, mean fixation duration, and time to first fixation — are empirically stable across the four cells, including the affected one, and their conclusions are independently reproduced by the trial-level mixed-effects models. We do not claim that these measures are immune to processing differences merely by virtue of being ratios or latencies — the distractor ratio shares the same ratio form yet is badly distorted in one cell — but rather that the specific measures we rely on are demonstrably unaffected here, as the mixed-effects reanalysis confirms. Fully repairing the raw-count families would require reprocessing the raw gaze recordings through a single uniform pipeline; because the substantive results do not depend on those families, we treat this as a limitation bounding the interpretation of the raw-count metrics rather than as work required to support the study's conclusions, which rest entirely on the count-insensitive measures. Finally, the sample comprised students from two disciplines at a single institution; generalisation to other disciplines, institutions, and training stages requires replication.

### Future directions

Two extensions follow directly. First, collecting behavioural responses, namely participants’ actual interpretive judgements for each image, would allow a proper test of whether attention to critical regions predicts correct interpretation, shifting the design from attention alone to the attention–accuracy relationship. Second, a longitudinal design that follows the same students from early training through accumulating clinical exposure would directly test whether the disciplinary perceptual advantage absent here emerges later, and, if so, how much and what kind of practice is required, a question the cross-sectional design cannot answer.

## Conclusions

In a within-stimulus 2 × 2 mixed-design study, early vocational health students showed no advantage in attending to the clinically critical regions of their own discipline's images compared with students for whom those images were unfamiliar. Visual attention was governed by stimulus identity, not by viewer discipline, year of study, academic standing, or cognitive style. The only large ‘effects’ on raw-count metrics were identified as measurement artefacts and excluded. The findings argue against the assumption that disciplinary perceptual expertise is already established at this training stage and support its characterisation as an outcome of accumulated, deliberate clinical practice rather than of programme enrolment. For educators, the practical implication is that visual-scanning strategies should be taught explicitly and early, rather than assumed, while bearing in mind that directing attention is not the same as building competence.

## Data Availability

The raw data supporting the conclusions of this article will be made available by the authors, without undue reservation.

## References

[1] Just MA Carpenter PA. A theory of reading: from eye fixations to comprehension. Psychol Rev. (1980) 87(4):329–54. 10.1037/0033-295X.87.4.329 7413885

[2] Rayner K. Eye movements and attention in reading, scene perception, and visual search. Q J Exp Psychol. (2009) 62(8):1457–506. 10.1080/17470210902816461 19449261

[3] Specian Junior FC Litchfield D Sandars J Cecilio-Fernandes D. Use of eye tracking in medical education. Med Teach. (2024) 46(11):1502–9. 10.1080/0142159X.2024.2316863 38382474

[4] Wilbanks BA McMullan SP. A review of measuring the cognitive workload of electronic health records. Comput Inform Nurs. (2018) 36(12):579–88. 10.1097/CIN.0000000000000469 30134256

[5] Kok EM Jarodzka H. Before your very eyes: the value and limitations of eye tracking in medical education. Med Educ. (2017) 51(1):114–22. 10.1111/medu.13066 27580633

[6] Sugimoto M Oyamada M Sato M. Eye-tracking in nursing simulation: a scoping review of educational design and assessment. Teach Learn Nurs. (2026) 21(2):e876–84. 10.1016/j.teln.2025.11.033

[7] Sáiz-Manzanares MC Marticorena-Sánchez R Martín Antón LJ González-Díez I Carbonero Martín MÁ. Using eye tracking technology to analyse cognitive load in multichannel activities in university students. Int J Hum–Comput Interact. (2024) 40(12):3263–81. 10.1080/10447318.2023.2188532

[8] Aksu ŞH Çakit E Dağdeviren M. Mental workload assessment using machine learning techniques based on EEG and eye tracking data. Appl Sci. (2024) 14(6):2282. 10.3390/app14062282

[9] Lorenz J Zevano J Otto N Schneider B Papan C Missler M. Looking at social interactions in medical education with dual eye-tracking technology: a scoping review. MedEdPublish. (2024) 14:215. 10.12688/mep.20577.2 39931308 PMC11809160

[10] Sáiz-Manzanares MC, Rodríguez-Díez JJ, Díez-Pastor JF, Rodríguez-Arribas S, Marticorena-Sánchez R, Ji YP. Monitoring of student learning in learning management systems: an application of educational data mining techniques. Appl Sci. (2021) 11(6):2677. 10.3390/app11062677

[11] Stuijfzand BG Van Der Schaaf MF Kirschner FC Ravesloot CJ Van Der Gijp A Vincken KL. Medical students’ cognitive load in volumetric image interpretation: insights from human–computer interaction and eye movements. Comput Human Behav. (2016) 62:394–403. 10.1016/j.chb.2016.04.015

[12] Sondermann C Huff M Merkt M. Distracted by a talking head? An eye tracking study on the effects of instructor presence in learning videos with animated graphic slides. Learn Instr. (2024) 91:101878. 10.1016/j.learninstruc.2024.101878

[13] Zhang S De Koning BB Paas F. Effects of mouse pointing on learning from labeled and unlabeled split-attention materials: an eye-tracking study. Comput Human Behav. (2023) 143:107673. 10.1016/j.chb.2023.107673

[14] Sweller J. Measuring cognitive load. Perspect Med Educ. (2018) 7(1):1–2. 10.1007/S40037-017-0395-4 29305818 PMC5807263

[15] Mayer RE. Cognitive theory of multimedia learning. In: Mayer RE, editor. The Cambridge Handbook of Multimedia Learning. 2nd ed. Cambridge: Cambridge University Press (2014). p. 43–71.

[16] Tokuno J Carver TE Fried GM. Measurement and management of cognitive load in surgical education: a narrative review. J Surg Educ. (2023) 80(2):208–15. 10.1016/j.jsurg.2022.10.001 36335034

[17] Sáiz-Manzanares MC Marticorena-Sánchez R Escolar-Llamazares M-C Martín-Antón LJ Velasco-Saiz R. Advanced learning technologies and integrated multichannel eye tracking for monitoring self-regulated learning. Think Skills Creat. (2025) 58:101938. 10.1016/j.tsc.2025.101938

[18] Johannessen E Szulewski A Radulovic N White M Braund H Howes D. Psychophysiologic measures of cognitive load in physician team leaders during trauma resuscitation. Comput Human Behav. (2020) 111:106393. 10.1016/j.chb.2020.106393

[19] Clauss K Bardeen JR Gordon RD Daniel TA. Increasing cognitive load attenuates the moderating effect of attentional inhibition on the relationship between posttraumatic stress symptoms and threat-related attention bias variability. J Anxiety Disord. (2021) 81:102416. 10.1016/j.janxdis.2021.102416 33991820

[20] Wilbanks BA Aroke E Dudding KM. Using eye tracking for measuring cognitive workload during clinical simulations: literature review and synthesis. Comput Inform Nurs. (2021) 39(9):499–507. 10.1097/CIN.0000000000000704 34495011

[21] Holmqvist K Andersson R. Eye Tracking: A Comprehensive Guide to Methods, Paradigms and Measures. 2nd ed. Lund: Lund Eye-Tracking Research Institute (2017).

[22] Eckstein MK Guerra-Carrillo B Miller Singley AT Bunge SA. Beyond eye gaze: what else can eyetracking reveal about cognition and cognitive development? Dev Cogn Neurosci. (2017) 25:69–91. 10.1016/j.dcn.2016.11.001 27908561 PMC6987826

[23] Liberman L Dubovi I. The effect of the modality principle on learning with virtual reality: an eye-tracking and electrodermal activity study. J Comput Assist Learn. (2024) 40(2):545–58. 10.1111/jcal.12763

[24] Marquart G Cabrall C De Winter J. Review of eye-related measures of drivers’ mental workload. Procedia Manuf. (2015) 3:2854–61. 10.1016/j.promfg.2015.07.783

[25] Argunsah H Altıntaş L Şahiner M. Eye-tracking insights into cognitive strategies, learning styles, and academic outcomes of turkish medicine students. BMC Med Educ. (2025) 25:276. 10.1186/s12909-025-06855-y 39979922 PMC11843750

[26] Szulewski A Roth N Howes D. The use of task-evoked pupillary response as an objective measure of cognitive load in novices and trained physicians: a new tool for the assessment of expertise. Acad Med. (2015) 90(7):981–7. 10.1097/ACM.0000000000000677 25738386

[27] Carneiro Queiroz MG Specian Junior FC Hamamoto Filho PT Santos TM Schauber SK Woltman AM. Comparing the cognitive workload of very short answer questions and multiple-choice questions using eye tracking. Med Educ Online. (2026) 31(1):2621434. 10.1080/10872981.2026.2621434 PMC12849799 41588719

[28] Huangfu Q Deng T Guo Y Li Y Feng R Wang Z. Prior knowledge interacts with the effects of pre-questions and feedback types on learning from videos: eye-tracking and cognitive load evidence. Br J Educ Technol. (2026) 57(4):1115–39. 10.1111/bjet.70046

[29] Itti L Koch C. Computational modelling of visual attention. Nat Rev Neurosci. (2001) 2(3):194–203. 10.1038/35058500 11256080

[30] Ayres P Sweller J. The split-attention principle in multimedia learning. In: Mayer RE, editor. The Cambridge Handbook of Multimedia Learning. 2nd ed. Cambridge: Cambridge University Press (2014). p. 206–26.

[31] Dias RD Ngo-Howard MC Boskovski MT Zenati MA Yule SJ. Systematic review of measurement tools to assess surgeons’ intraoperative cognitive workload. Br J Surg. (2018) 105(5):491–501. 10.1002/bjs.10795 29465749 PMC5878696

[32] Paas F Tuovinen JE Tabbers H Van Gerven PWM. Cognitive load measurement as a means to advance cognitive load theory. Educ Psychol. (2003) 38(1):63–71. 10.1207/S15326985EP3801_8

